# Effects of Extrinsic Mortality on the Evolution of Aging: A Stochastic Modeling Approach

**DOI:** 10.1371/journal.pone.0086602

**Published:** 2014-01-21

**Authors:** Maxim Nikolaievich Shokhirev, Adiv Adam Johnson

**Affiliations:** 1 Program in Bioinformatics and Systems Biology, University of California San Diego, La Jolla, California, United States of America; 2 Physiological Sciences Graduate Interdisciplinary Program, University of Arizona, Tucson, Arizona, United States of America; University of Lausanne, Switzerland

## Abstract

The evolutionary theories of aging are useful for gaining insights into the complex mechanisms underlying senescence. Classical theories argue that high levels of extrinsic mortality should select for the evolution of shorter lifespans and earlier peak fertility. Non-classical theories, in contrast, posit that an increase in extrinsic mortality could select for the evolution of longer lifespans. Although numerous studies support the classical paradigm, recent data challenge classical predictions, finding that high extrinsic mortality can select for the evolution of longer lifespans. To further elucidate the role of extrinsic mortality in the evolution of aging, we implemented a stochastic, agent-based, computational model. We used a simulated annealing optimization approach to predict which model parameters predispose populations to evolve longer or shorter lifespans in response to increased levels of predation. We report that longer lifespans evolved in the presence of rising predation if the cost of mating is relatively high and if energy is available in excess. Conversely, we found that dramatically shorter lifespans evolved when mating costs were relatively low and food was relatively scarce. We also analyzed the effects of increased predation on various parameters related to density dependence and energy allocation. Longer and shorter lifespans were accompanied by increased and decreased investments of energy into somatic maintenance, respectively. Similarly, earlier and later maturation ages were accompanied by increased and decreased energetic investments into early fecundity, respectively. Higher predation significantly decreased the total population size, enlarged the shared resource pool, and redistributed energy reserves for mature individuals. These results both corroborate and refine classical predictions, demonstrating a population-level trade-off between longevity and fecundity and identifying conditions that produce both classical and non-classical lifespan effects.

## Introduction

The evolutionary theories of aging attempt to explain why natural selection would favor the almost ubiquitous evolution of senescence, a process which markedly decreases Darwinian fitness. Classical theory can be subdivided into three distinct yet analogous perspectives – Medawar's “mutation accumulation,” Williams's “antagonistic pleiotropy,” and Kirkwood's “disposable soma” theories [1]–[3]. The “mutation accumulation” theory posits that, since extrinsic mortality (e.g., predation and disease) is typically high in the wild, few animals will survive long enough to exhibit senescence and the force of natural selection will decline with age. As such, late-acting deleterious mutations will accumulate over time and passively lead to the development of aging [4]. It should be noted that, while old age is undoubtedly rare for many species (e.g., wild mice) [5], there are notable exceptions (e.g., naked mole rats [6] and blind cave salamanders [7]). Moreover, senescence has been observed in the wild for many species of mammals, birds, other vertebrates, and insects [8]. The “antagonistic pleiotropy” theory supposes that senescence evolved due to the active selection of pleiotropic genes, which are beneficial early in life and harmful later in life [9]. The similar yet more mechanistic “disposable soma” theory argues that, since resources are often limited and the force of natural selection generally declines with age, an organism that allocates more energy into early reproduction versus long-term somatic maintenance will be more successful. Thus, according to this theory, aging results from a lack of investment in anti-aging mechanisms [10], [11]. Furthermore, according to the “antagonistic pleiotropy” and “disposable soma” theories, evolutionary trade-offs should exist between lifespan and reproduction [1]–[3].

These three classical theories all predict that extrinsic mortality levels should be inversely correlated with evolved lifespan. Non-classical theories, in contrast, predict that increased extrinsic mortality could select for the evolution of longer lifespans. Field observations have shown that opossums from an insular, low-predation population exhibit reduced litter sizes and delayed senescence compared to a mainland, high predation population [12]. Daphnia from temporary ponds were observed to experience higher rates of mortality than those from permanent lakes [13]. Concomitant with this, Daphnia from temporary ponds exhibited shorter lifespans and faster juvenile growth compared to those from permanent lakes [14]. In *Drosophila melanogaster*, a decrease in longevity and earlier peak fecundity can be directly selected for by increasing extrinsic mortality levels [15]. Garter snakes from low extrinsic mortality environments were found to have longer lifespans than those from high mortality environments [16] and annual fish of the genus *Nothobranchius* from a dry region exhibited shorter captive lifespans than those from a humid region [17]. Furthermore, numerous adaptations that result in decreased extrinsic mortality – such as arboreality, wings, shells, and larger brains – are associated with increased longevity [18], [19]. Other evidence comes from a deterministic, computational model developed by Drenos and Kirkwood, which found that higher levels of extrinsic mortality results in the reallocation of energy from somatic maintenance to reproduction [20]. The reverse was also reported in a model published by Cichoń, which found that low rates of environmentally-induced mortality promoted allocation of energy to repair instead of reproduction [21]. It was also shown in a separate theoretical optimization model that the evolution of shorter lifespan in insect workers is favored by risky environments [22].

Although corroborative of a role for extrinsic mortality in driving the evolution of aging, other studies challenge the classical predictions. In guppies derived from natural populations, fish from high predation localities exhibited earlier maturation ages, enhanced swimming performance, and delayed senescence compared to fish from low predation sites [23]. In the nematode *Caenorhabditis remanei*, increasing random versus condition-dependent extrinsic mortality had differential effects on evolved lifespan: High random mortality selected for a shorter lifespan while high condition-dependent mortality (induced by heat stress) engendered nematodes that were longer lived. No obvious effects on reproduction were observed for nematodes subjected to either type of mortality [24], possibly due to a subpopulation of heat-stressed nematodes benefiting from the death of their neighbors. Auxiliary support comes from a deterministic, mathematical model developed by Abrams, which theorized that, depending on changes in density dependence and age-class specificity of externally introduced mortality, aging may evolve to increase, decrease, or remain unaffected [25]. While the modeling approach reveals that age-specific effects can produce a decreased rate of senescence in response to density-dependent mortality, underlying heterogeneity of populations, population instability, shared energy interdependence, starvation effects, and energy associated with mating were not considered [25]. Indeed, in a recent paper modeling the evolution of aging, population heterogeneity was shown to be an important feature of the population response [26]. These parameters were also not considered in a follow-up, deterministic model by Williams and Day, which showed that the internal condition of an individual can affect selection against physiological deterioration in response to interactive mortality sources [27].

Both modeling and empirical data show that extrinsic mortality can lead to the evolution of either increased or decreased intrinsic mortality. Outside of age-class specificity [25] and the internal condition of an individual [27], the factors that predispose a population to evolve one way or another remain largely unknown. In addition, typically, models of life histories represent homogenous populations under static conditions for which reproduction and survival are modeled by age-dependent differential equations that can be solved to find conditions of optimal net reproductive rates. However, natural populations exhibit features such as finiteness, stochasticity, interdependence, and heterogeneity, allowing for counterintuitive effects. It has been previously shown that guppies subjected to higher levels of predation exhibit delayed senescence [23] and have higher per-capita resource availability than guppies from low-predation localities [28]. We therefore hypothesized that non-classical increases in longevity can naturally arise when energy becomes plentiful due to an increase in extrinsic mortality (i.e., when energy that would otherwise be used by neighbors is freed up for consumption by the remaining population). Furthermore, we hypothesized that this non-classical effect would only arise under specific model conditions, since the majority of the published data suggest that the classical response is much more common. Therefore, we implemented an energy allocation model in the context of a non-stable, finite, stochastic, and interdependent population. To test this hypothesis, we constructed an agent-based computational model in which each individual is subject to shared resource availability, sexual reproduction, intrinsic and evolvable allocation of resources toward repair and maturation, density-dependent death from predation, intrinsic evolvable death times and extrinsic death from starvation. Our modeling approach is powerful because it stems from first-principles about energy allocations and because we are able to model each individual explicitly, allowing us to capture features of natural populations. The incorporation of finiteness, stochasticity, population heterogeneity, and interdependence uniquely distinguishes our model from previously published models investigating the evolution of aging [20]–[22], [25], [27].

To test which (if any) conditions can lead to the evolution of an increase or decrease in average longevity, we used simulated annealing optimization [29] to determine which global parameters (e.g., food generation rate, maturation efficiency, mating cost and threshold) bias populations toward the evolution of higher or lower intrinsic mortality as a function of extrinsic death. Using this unbiased parameterization approach, we found that longer lifespans should evolve naturally in the presence of increasing predation if the cost of mating is relatively high and if energy is generated in excess. Conversely, we found that the classical, decreased investment in aging repair was most dramatic when mating costs were relatively low and when food was relatively scarce. By employing simulated annealing to identify these conditions, our study largely avoided biases caused by manual model parameter selection.

## Methods

**Figure 1:**
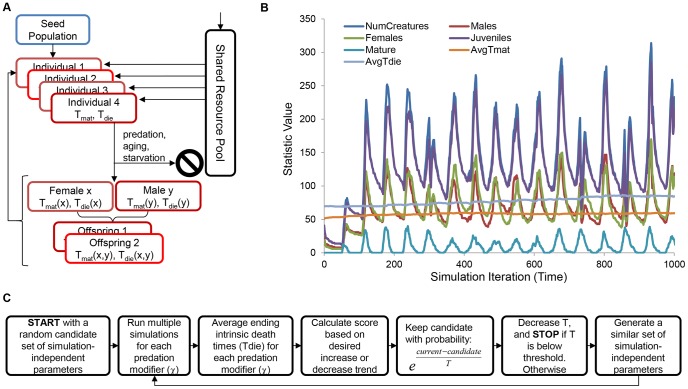
Overview of the modeling and optimization procedures. An agent-based stochastic model was implemented in which individuals invest energy foraged from a common pool toward maturation, metabolism, mating, and maintenance and are subject to random, density-dependent predation, starvation, and aging. Maturation and intrinsic death times are inheritable traits used to determine maturation, and maintenance costs. A flowchart depicts the simulations scheme (**A**). Sample simulation solution depicting changes in observed statistics with time is shown (**B**). To find appropriate values for six simulation-invariant parameters for the classical and non-classical evolutionary response to increased predation a simulated annealing optimization approach was used (**C**). See methods for model and optimization details.

We studied the evolution of aging in populations using a stochastic agent-based iterative computational model subject to randomness associated with foraging, mate selection, mating success, predation, inheritance of intrinsic longevity and maturation times, and starvation. In addition to inherent random fluctuations, populations were allowed to evolve over time as individuals with more favorable energy allocation mechanisms were more likely to pass on their intrinsic longevity and maturation times to their offspring (Fig. 1). We derive the computational model, and describe the methods we used to solve and fit the model in the sections below. We have included a summary of all model parameters in Table 1.

**Table 1:** Summary of key model parameters and quantities.

Parameter	Description	Value	Justification/Explanation
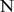	Population size modifier	50	Chosen to be high enough to avoid extinction from random fluctuations. Actual population size is stochastic and depends on other parameters.
***runTimes***	Number of independent simulation runs averaged	400	Value was chosen to be high enough to clearly separate steady-state simulation values between simulation conditions.
**D**	Simulation duration	300,000 iteration rounds	Empirically selected to produce stable simulation results.
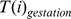	Gestation time	1 iteration rounds	Assumed to be approximately 1/10 of the maturation time or 1/100 of the lifespan
***NumOffspring***	Number of offspring	1 to 2 offspring (1–8 offspring)	Arbitrary, but does not affect results qualitatively. (Alternate: 1+8[(a(i)−T_mat_(i))/(T_die_(i)−T_mat_(i))])
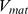	“evolvability” of the maturation age	0.2	Chosen to be high enough to ensure convergence of simulations toward stable values in D simulation rounds.
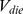	“evolvability” of the intrinsic aging death age	0.2	Chosen to be high enough to ensure convergence of simulations toward stable values in D simulation rounds.
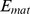	Energy of mature individuals	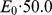	We assumed that mature individuals are roughly 50 times larger than newborn individuals.
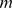	Adult metabolic cost	1.0	Scaled to be the highest energy cost of mature individuals. Shared energy reserves used this value as a scaling factor so it is mostly arbitrary.
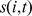	Fractional size of individual *i*	[ 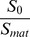 −1]	Used to scale energy costs for non-mature individuals.
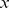	Predation modifier	0.0025,0.005, 0.01,0.02,0.04	Independent variable affecting the probability of age-independent death from predation.
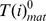	Initial maturation age	45 to 55 iteration rounds	Set to be initially high to avoid population collapse due to prohibitive costs.
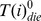	Initial intrinsic aging death age	65 to 75 iteration rounds	Set to be initially low to avoid population collapse due to prohibitive costs.
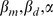	Uniformly distributed random variables	[0–1]	These stochastic variables are used to randomly adjust the inherited mating time, death time, and foraging rate.
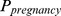	Intrinsic probability of pregnancy	[0–1] or (0.5)	Decreases with age: 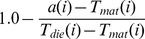 (Alternate: constant at 0.5)
Parameters optimized by simulated annealing (allowed to vary within a range)			
ε	Starvation modifier	0.5 to 3.0	Most deaths are caused by starvation for ε = 0.5 while, most deaths are not caused by starvation for ε = 3.0 when the population size is ∼ N.
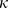	Growth efficiency	0.01 to 0.25	The fraction of foraged energy converted to mass (increasing from 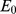 toward 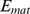 ).
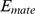	Mating energy	(0.01 to 0.5) 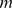	Energy expended on mating.
*mateThreshold*	Mating energy threshold	(0.01 to 0.5) 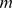	Energy needed to be eligible for mating.
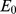	Initial energy of individuals	0.001 to 0.1	Starting energy of newborn individuals.
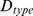	Death cost function type	Six possible types (eq. 8)	[0 = Sigmoidal Low, 1 = Linear Low, 2 = Asymptotic Low, 3 = Sigmoidal High, 4 = Linear High, 5 = Asymptotic High].

Populations were modeled explicitly using stochastic agents to represent individuals subject to shared resources, mating, and both extrinsic and intrinsic death.

### Modeling evolving populations

In order to explore how predation-mediated energy availability may bring about the evolution of increased lifespans (non-classical) or decreased lifespans (classical), we derived a stochastic agent-based computational model starting from energy allocation assumptions. To start, we assumed that each individual, *i*, keeps track of energy level (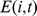), age (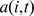), gender (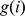), chosen mate, (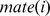), as well as an inherited age to maturation (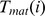) and inherited intrinsic lifespan (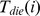). We assumed that available total forage energy (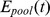) is kept in a common energy pool and generated at a rate proportional to the population size modifier (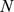), the adult metabolic energy cost (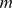), and a starvation modifier (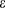):
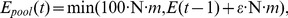
(1)


We also assumed that the shared energy pool has a maximum capacity of 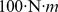, representing saturation of the food source in the environment. In order to calculate the flow of energy through the model, we assumed that each individual *i*, calculates energy costs (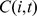) for repair, maturation, and metabolism for each model iteration t:
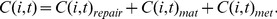
(2)


We assumed a creature *i* grows in size while maturing and the size is proportional to its energy by a factor (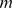). Newborn individuals start with energy 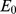 (equivalent to 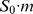) and grow by a fraction proportional to its current size until reaching the mature energy level, 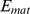 (equivalent to 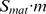):
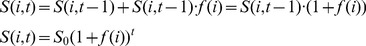
(3)


We then solved for 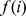 by substituting the known boundary conditions into (3):
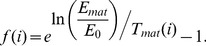
(4)


Using (3) we calculated the fractional size of an individual at any point in time, 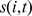 as:
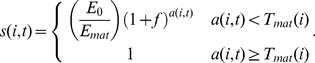
(5)


By assuming that the energy required to grow at each iteration is proportional to the current size, and allowing for an energy conversion modifier (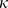), we calculated the per-iteration maturation energy cost as:
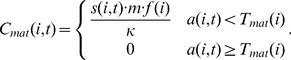
(6)


Next, we assumed that aging is caused by non-ideal iterative repair, which incurs a per-iteration cost, proportional to the relative size (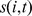) of each individual and a function of the inherited time to death (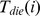) of each individual:
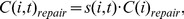
(7)where the time-independent cost function (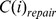) can have one of six forms:
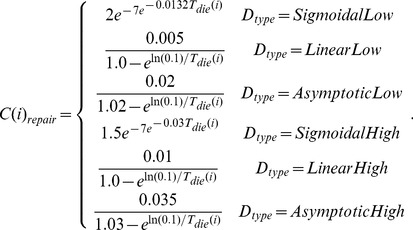
(8)


While it is possible that the per-iteration cost of aging will change with age, energy availability, gender, and other characteristics, we were unaware of a biologically tested general model for the per-iteration cost of repair. Instead, we modeled the per-iteration cost as one of six functions of the intrinsic death time, as shown in eq. (8). However, we allowed the repair cost function to vary in order to avoid biasing the behavior of the model toward the evolution of higher or lower intrinsic death times, instead treating the shape of the repair function as an independent variable that may take on linear, sigmoidal, or asymptotic shapes (Fig. S1). Furthermore, we allowed for both a relatively “cheap” and a relatively “expensive” version of each shape to improve the flexibility of model fitting.

Finally, we calculate the energy required for per-iteration metabolic expenditures, which is assumed to be constant and proportional to the current relative size of each individual:
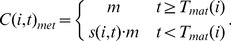
(9)


After calculating the immediate energy expenditures, each individual determines the energy foraged (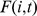) from available common food in a “greedy” fashion (in random order each iteration round), subject to an intrinsic stochastic foraging capacity (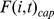) and an energy storage maximum set to approximately five iterations of maximum foraging (i.e., an individual requires approximately five iterations to fully replenish its energy supply in the absence of energy expenditure):
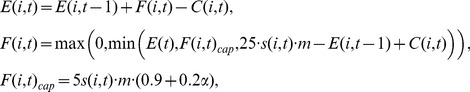
(10)where 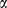 is a uniformly distributed random number from 0 to 1 added to decrease the chance of extinction by starvation which can arise due to the discrete simulation methodology. Constants in eq. (10) were manually selected to ensure that foraging would result in a mean increase in stored energy under typical simulation conditions. We assumed density-independent foraging for several reasons. First, we assumed that energy availability decreases with trophic level (i.e., vegetation is ubiquitous, while predators must search for prey). Second, we wanted to keep the model as simple as possible in order to simplify analyses.

Finally, the foraged energy is removed from the common pool of energy,
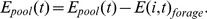
(11)


At this point, if 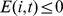, the individual was marked as “starved” and removed from further simulation (died). In addition to death from starvation, individuals are subjected to density dependent predation. The probability that each individual dies from predation during each simulation round, t, was assumed to be:
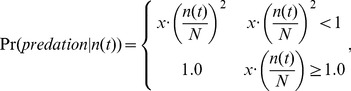
(12)where 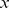 is the predation modifier parameter used to linearly affect the magnitude of predation-dependent death. If the individual was randomly selected to die from predation, it was marked as “eaten” and removed from subsequent simulation iterations. Finally, if an individual reached or surpassed its old age limit (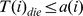), it was removed from the population and marked as dead from intrinsic “aging” death.

After accounting for intrinsic energy costs and death effect, surviving, mature females were mated with surviving mature males. Since we assumed that the total lifespan of individuals in the model is relatively short (on the order of 100 iteration rounds), we assumed for simplicity that all eligible individuals have sufficient time to find suitable mates during each iteration round. We imposed energy requirement for individuals to engage in mating behavior: both females and males were required to be non-starving (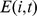
>*mateThreshold*) and females were required to be non-gestating (not currently mated). Mating resulted in the expenditure of energy, 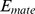 for both sexes regardless of outcome and represented the energy required to find, attract and/or copulate with a mate. The probability that female *i* becomes pregnant was defined to decrease linearly as a function of age after maturation according to:
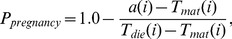
(13)where 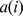 is the current age, 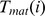 is the maturation age, and 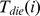 is the age at which the female will die from old age (intrinsic death time). We assumed that male fertility does not depend on age for simplicity. Each eligible female was allowed to mate with each eligible male in a randomized fashion until she became ineligible (e.g., successful mating or starving), or until no eligible bachelors existed (e.g., all had insufficient energy). Finally, all females that finished gestating (survived since last iteration) gave birth to between one and two offspring (to avoid gender bias, we assumed no cost associated with giving birth). When testing the effect of increasing fertility as a function of age, the probability of pregnancy was set to 0.5 instead of eq. (13), but the number of offspring increased as a function of age according to:
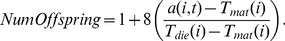
(14)


For each offspring z, maturation age 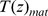, and intrinsic death age, 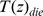, were assumed to be inherited from the mother *j* and father *k* according to the following linear probability rules:
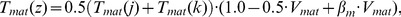
(15)

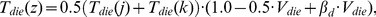
(16)and where 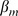 and 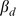 are random uniformly distributed variables used to mimic mutation and 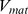 and 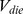 are “evolvability” constants that determine the degree of variability of each parameter. Therefore, the intrinsic maturation and death times (and therefore the daily repair and maturation costs) are inherited from both parents and further modified by a random multiplier representing mutation. All offspring start out with age, 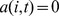, and equal probability of being female or male. Initially, each simulation is started with equal numbers of newborn males and females. Since simulations include stochastic fluctuations in the amount of energy foraged, predation, and order, we iteratively computed 300,000 simulation iterations following the logic described above and averaged across 400 independent simulations to ensure even sampling. We selected 300,000 iteration steps to ensure that the average inherited maturation and death times stabilized.

### Optimizing global model parameters with simulated annealing

While individuals in each simulation keep track of evolving parameters for the amount of energy they devote to repair and maturation (used to calculate intrinsic 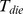 and 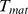), several simulation invariant parameters can bias the population toward the evolution of an increased or decreased average repair or maturation rate. For example, the amount of energy generated per simulation iteration should affect the average foraging amounts of individuals at a given density. Furthermore, since conclusions drawn from mathematical models may be non-general or subject to parameter bias, we decided to define a range of values for several simulation-invariant parameters and used a simulated annealing approach [29] to identify sets of parameter values that favor the classical (i.e., decrease in repair with increased predation) or non-classical aging strategies (i.e., increase in repair with increased predation). Simulated annealing consisted of simulations using a specific set of simulation-invariant parameters which were evaluated for cases of increasing extrinsic mortality from predation: (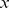 = 0.0025, 0.005, 0.01, 0.02, and 0.04). At the end of each simulation, average 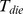 times were calculated for eight independent simulations, averaging the last 1,000 recorded population average 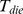 times to improve the signal to noise ratio. Next, a score was assigned for the current set of simulation-invariant parameters based on whether we were biasing toward a non-classical:
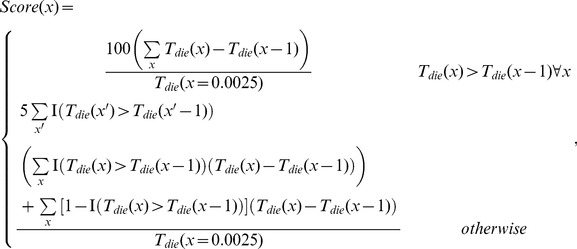
(17)or classical response:
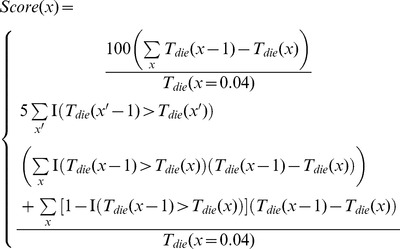
(18)


where 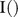 is the indicator function, and the score is improved if death times are observed to change in the desired fashion, and especially if the change is monotonic. After scores are calculated, a set of candidate parameters are kept with a probability that depends on the current annealing temperature 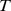 (an optimization parameter) and difference in scores according to:
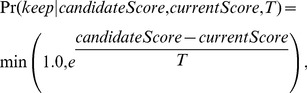
(19)


At the end of each annealing optimization round, the temperature is multiplied by 0.95, resulting in increasingly unlikely probabilities of keeping sets of poor parameters at later iterations. The initial and ending temperatures were empirically chosen to be 100 and 0.5, respectively, resulting in 104 annealing rounds. Candidate parameter sets leading to unstable simulations (all eight went extinct) were discarded without temperature changes. At the end of the simulated annealing procedure, the set of parameters that resulted in the highest recorded score were selected for more detailed simulations and analysis. For a set of simulation-invariant parameters and their allowed ranges see Table 1. All java code is available free of charge for download at https://github.com/Shokhirev/EvolutionOfAging.

## Results

To explore which conditions can lead to a non-classical or classical response to increased extrinsic mortality, we implemented a computational model of an evolving population of individuals subject to shared resources, sexual reproduction, extrinsic density-dependent death from predation, extrinsic death from starvation, and intrinsic inheritable somatic repair rates (Fig. 1). Adopting an energy investment model, individuals are subject to a constant, size-dependent metabolic cost, age-independent inherited somatic repair cost, and an age-dependent inherited maturation cost, along with both an energy cost and threshold required for mating. Furthermore, the probability of successful reproduction is assumed to be linearly dependent on the maternal age after maturation with highest and lowest probability of mating fixed at the inherent 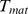 (maturation age) and 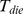 (longevity) values for each female, respectively. Fig. 1A shows a diagram summarizing our simulation procedure. Fig. 1B depicts typical low-predation population traces, which show characteristic density-dependent birth-death fluctuations. We next wanted to identify simulation parameters which bias populations to evolve higher or lower average intrinsic mortality times using a simulated annealing optimization approach.

### Simulated annealing reveals conditions favoring classical vs. non-classical responses to predation

**Figure 2:**
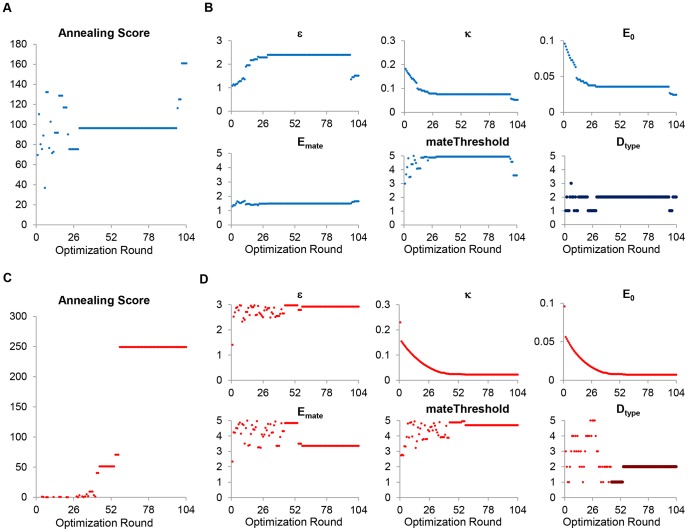
Classical and non-classical conditions identified by simulated annealing optimization. A simulated annealing optimization scheme was used to find values for six simulation-invariant parameters that would predispose populations toward either increased or decreased maintenance in response to increased extrinsic mortality. The fit score stochastically improved over the course of the optimization (**A and C**). The optimal starvation modifier (ε), growth efficiency (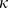), initial energy of individuals (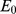), mating energy (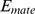), mating energy threshold (*mateThreshold*), and death cost function type (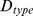) for the classical (**B**), and non-classical (**D**) effect are shown as a function of optimization duration. D_type_: 0 = Sigmoidal Low, 1 = Linear Low, 2 = Asymptotic Low, 3 = Sigmoidal High, 4 = Linear High, 5 = Asymptotic High. Colored dots indicate that the intrinsic death effect was monotonic.

To identify conditions that result in the evolution of shorter or longer average population lifespans in response to increased predation, we ran simulations with five different values for the predation modifier (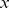 = 0.0025, 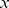 = 0.005, 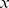 = 0.01, 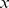 = 0.02, and 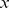 = 0.04). The predation modifier, 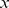, linearly scales the predation probability per individual per simulation iteration, for any given population density. Thus, as the predation modifier is increased, the odds of any individual dying from predation increase for a specific population density. We then used a simulated annealing approach (Fig. 1C) to find values of six simulation-invariant parameters resulting in the evolution of lower average intrinsic death times as a function of increasing predation (Fig. 2A and 2B, summarized in Table 1). Six parameters were optimized by simulated annealing: food turnover or starvation modifier (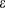) controls the number of deaths caused by starvation, mating energy (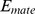) is the energy expended during mating, mating energy threshold (*mateThreshold*) is energy needed to eligible for mating, growth efficiency (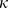) is the fraction of foraged energy converted to mass, 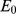 is the initial energy of newborn individuals, and 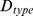 refers to the type of death cost function (sigmoidal, linear, and asymptotic low or high).

Specifically, lower average intrinsic death times evolved when energy (food) turnover was relatively low (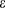 = 1.51), when the energy to mate was relatively cheap (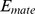 = 1.65), and when the energy required for mating was relatively moderate (*mateThreshold* = 3.58).

Next, we repeated the simulated annealing procedure (Fig. 2C and 2D) to determine if combinations of six simulation-invariant parameters can result in an opposite, non-classical effect (i.e., an increase in the average intrinsic time to die, 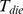, as a function of increasing predation modifier, 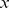). Results show that an increased lifespan can evolve and is favored by relatively abundant food conditions (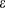 = 2.92), and both a relatively costly mating energy threshold (*mateThreshold* = 4.70) and mating cost (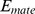 = 3.36). Interestingly, a low growth efficiency, low energy of newborn individuals, and a cheap asymptotic repair cost function were important for both the evolution of a decreased or increased average intrinsic death times (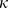 = 0.052, 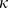 = 0.022, 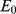 = 0.02, 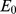 = 0.007, 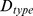 = 2 for both cases, respectively), suggesting that these parameters were primarily important for overall simulation stability and not necessary for producing differential responses to increased extrinsic mortality.

Interestingly, the shape of the repair cost function (i.e., how the cost of repair changes with the expected longevity), which was allowed to vary freely between six different types (Fig. S1) during the optimization process, revealed that both the increase and decrease response can evolve under various death cost assumptions (dark dots in Fig. 2B and 2D). However, while a decrease in intrinsic death times was seen for linear, asymptotic, or even sigmoidal cost functions, an increase was only seen with linear and asymptotic intrinsic death cost functions, suggesting that an increased lifespan is more likely to evolve if the increases in maintenance cost do not outpace the increase in lifespan.

### Increased predation can select for the evolution of shorter lifespans coupled to faster maturation or longer lifespans coupled to longer maturation times

**Figure 3:**
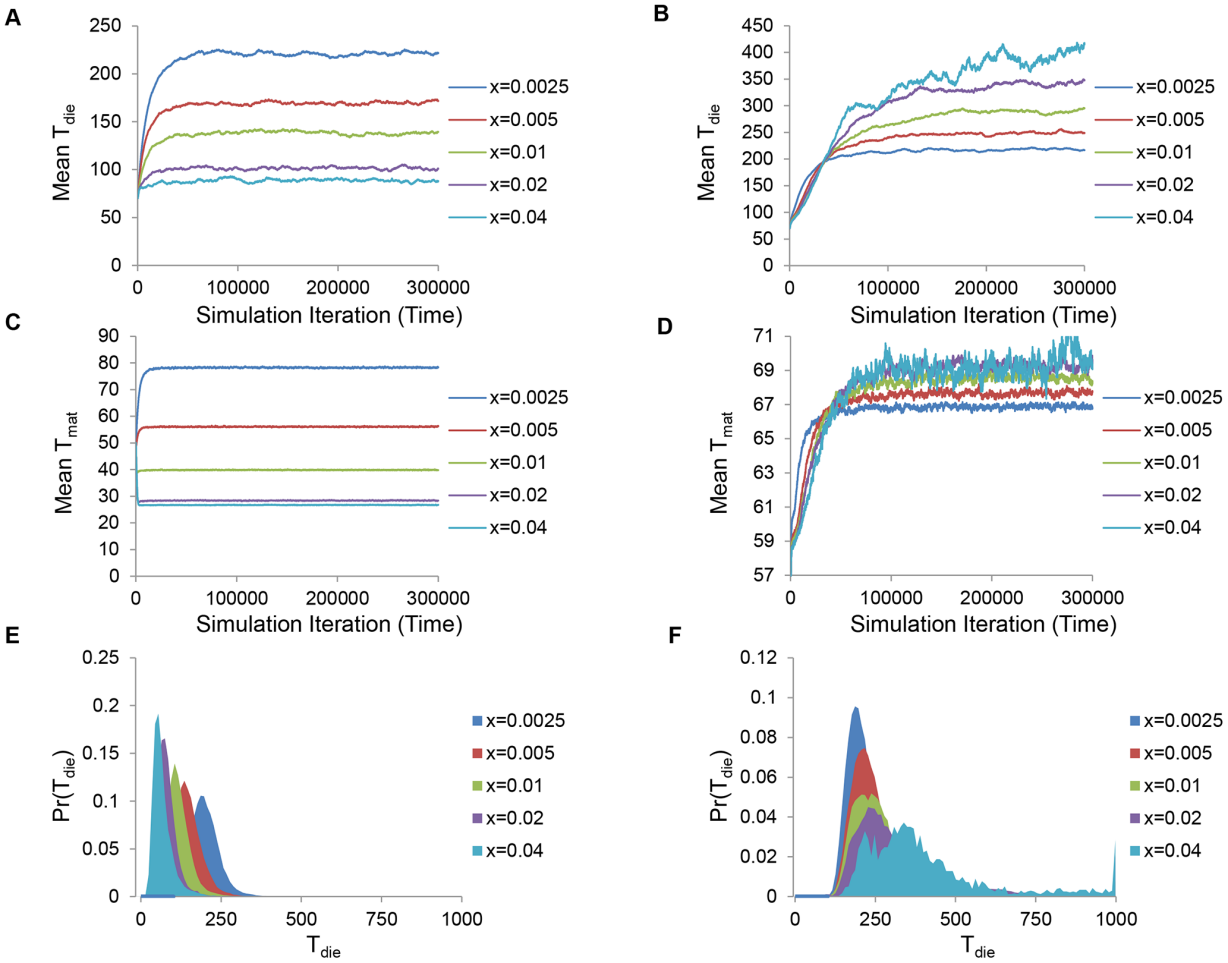
Disparate effects of high predation on the evolution of lifespan and maturation age. Under classical conditions of relatively low food availability and relatively inexpensive mating costs, increased values of predation modifier, x, caused mean T_die_ to decrease (**A**) and mean T_mat_ to decrease (**C**) over time. Conversely, under the non-classical conditions of relatively abundant food but relatively expensive mating costs, higher values of predation modifier, 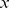, caused the mean T_die_ to increase (**B**) and the mean T_mat_ to increase (**D**). In (**E**) and (**F**), the population distribution of T_die_ is shown under classical (**E**) and non-classical (**F**) conditions at the end of 300,000 model iterations.

After identifying unique conditions impacting the evolution of aging, we analyzed how various statistics were affected by different levels of predation. We refer to classical conditions as conditions which favor a decrease in lifespan in response to higher predation. Conversely, we refer to non-classical conditions as conditions which result in the evolution of an increase in lifespan in response to higher predation. Under classical conditions, rising levels of predation selected for the evolution of shorter mean lifespans (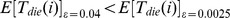) and earlier maturation ages (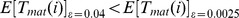), as exemplified in Fig. 3A and 3C, respectively. Conversely, non-classical conditions lead to an increase in mean lifespan (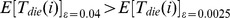) and mean maturation time (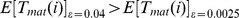), as shown in Fig. 3B, and Fig. 3D, respectively, although the increase in average maturation time was less dramatic. The entire distribution of population death times, (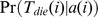), was shifted toward lower intrinsic death times for the classical condition (Fig. 3E), showing that under classical conditions the entire population reacted by deselecting investment into repair. On the other hand, under the non-classical conditions, heterogeneity in the population intrinsic death times (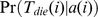) emerged with increasing extrinsic mortality (Fig. 3F), suggesting that population heterogeneity in intrinsic death times arises naturally in response to selective pressures from increased extrinsic mortality. However, high predation conditions leading to the evolution of decreased aging (longer mean 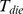) also resulted in population instability, with a large fraction of simulations becoming extinct under high predation conditions (Fig. S2). Interestingly, while only the highest predation condition resulted in drastic extinction for the increase case (Fig. S2B), the highest extrinsic mortality condition resulted in relatively stable populations under the classical conditions (Fig. S2A). In addition, while we assumed that fertility declined linearly with age (the probability of a successful mating decreases in the model), in some species, such as the Blanding's turtle, fertility can increase with age [30]. Therefore, we repeated the simulations but kept the probability of mating constant, while allowing the number of offspring to increase with age, thereby giving a reproductive advantage to older individuals. The observed trend persisted under increased fertility assumptions since increased extrinsic mortality lead to the evolution of longer or shorter lifespans under the non-classical or classical conditions, respectively (Fig. S3).

### Changes in evolved lifespan are matched by corresponding alterations in energy investment

**Figure 4:**
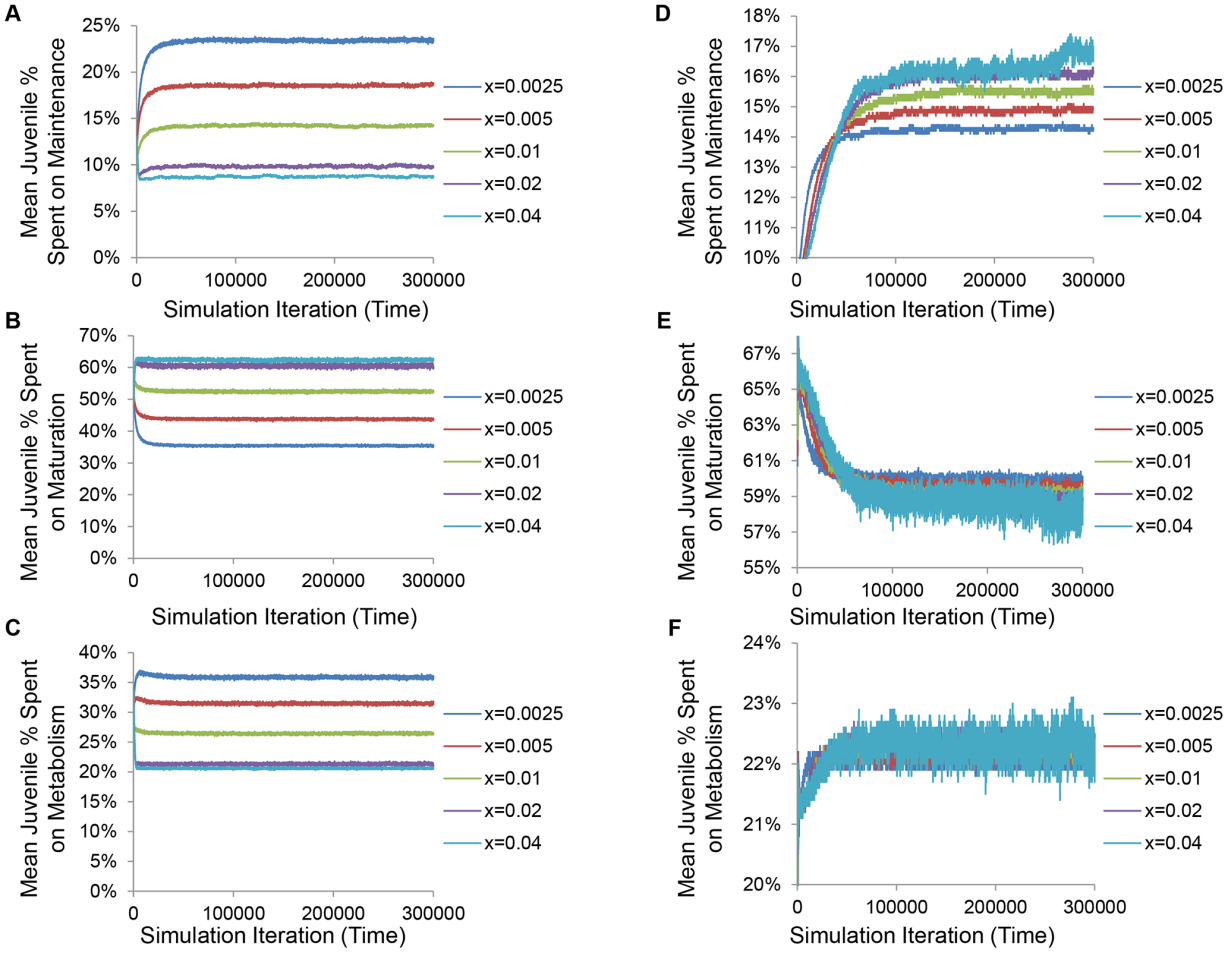
Changes in evolved lifespan and maturation age are accompanied by corresponding shifts in juvenile energetic investments. Under classical (**A–C**) and non-classical conditions (**D–F**), the percentage of per-iteration energy devoted to somatic maintenance, reproduction, and metabolism by juveniles is shown. In both cases, the majority of energy was devoted to reproduction, followed by metabolism, followed by somatic maintenance (**A–F**). Under classical conditions, rising levels of predation, 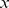, caused juveniles to invest less in somatic maintenance (**A**), more into early peak fertility (**B**), and less into metabolism (**C**). Under non-classical conditions, larger values of 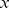 caused juveniles to devote less energy to early peak fertility (**E**) and more towards somatic maintenance (**D**). Investments in metabolism were comparable for various values of predation modifier, 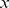 (**F**).

To gain insight into the mechanisms guiding these evolutionary changes, we investigated how different levels of predation impacted statistics related to population size as well as energy relocation and availability. Under classical conditions and rising values of extrinsic death, juveniles, who are subject to maturation costs in addition to metabolic and maintenance costs, invested relatively less energy in somatic maintenance (Fig. 4A), relatively more energy in early maturation (Fig. 4B), and relatively less energy in metabolism (Fig. 4C). Higher predation also caused mature individuals to invest less energy in somatic maintenance (Fig. S4A) and instead increase energy investments in mating and reproduction (Fig. S4B). These data show that, under conditions which select for the evolution of shorter lifespans in response to increased extrinsic mortality, individuals evolve to siphon energy away from somatic maintenance in order to invest in earlier maturation and mating.

Under non-classical conditions, however, higher levels of predation caused juveniles to invest relatively more energy into somatic maintenance (Fig. 4D) and relatively less energy into early maturation (Fig. 4E). Comparable fractional energetic investments in metabolism were observed regardless of the value of the predation modifier, 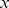 (Fig. 4F). Likewise, larger values of extrinsic mortality caused mature individuals to devote more energy to somatic maintenance (Fig. S4C). On the other hand, mature energy investment in mating and reproduction were reduced (Fig. S4D). Corroborative of the data presented in Figure 3, non-classical conditions encouraged individuals to reallocate energy away from early peak fertility and mating, instead investing in greater longevity.

**Figure 5:**
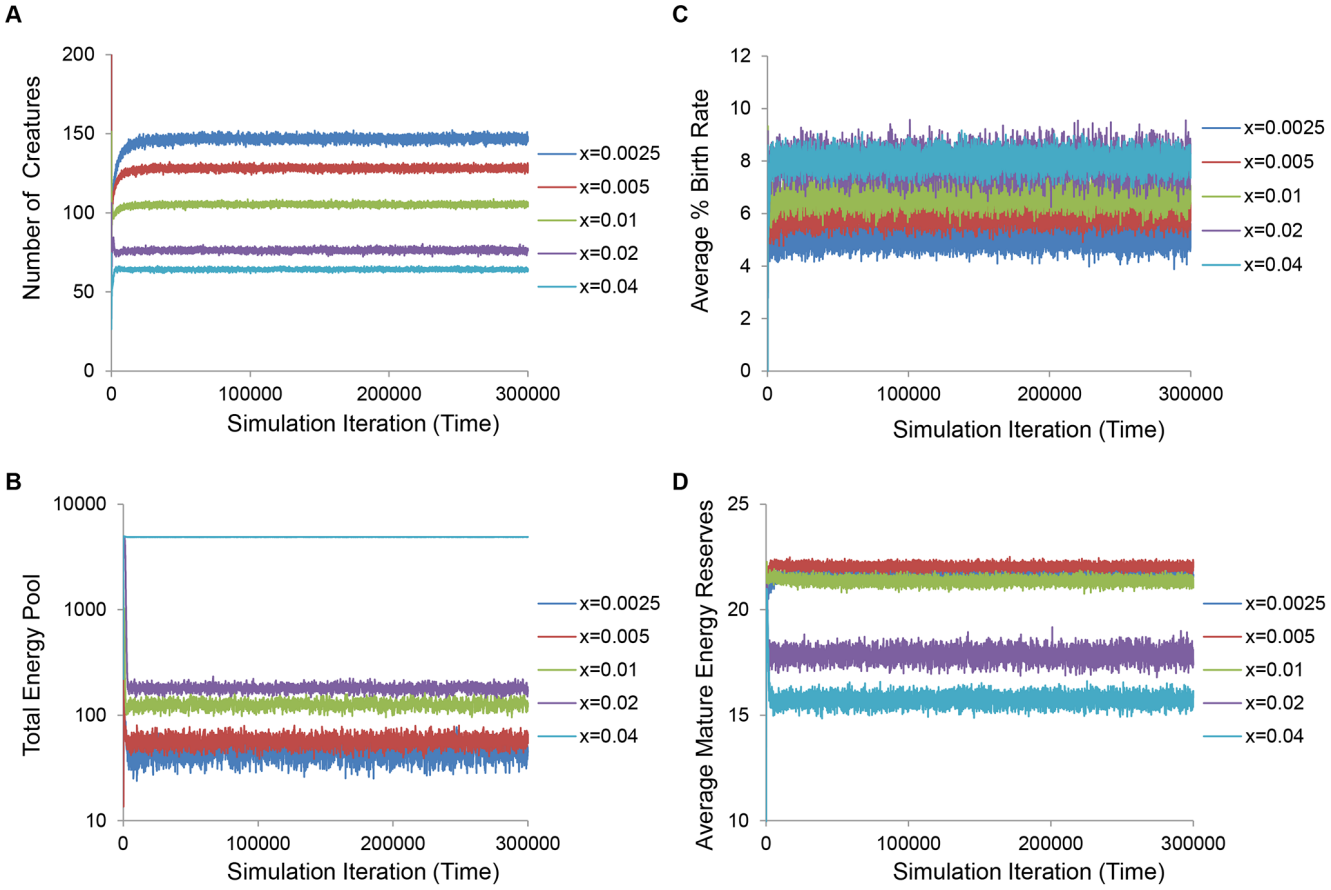
Higher predation impacts parameters related to density dependence under classical conditions. Under classical conditions, increased predation reduced population size (**A**) and enlarged the total energy pool that could be foraged (**B**). Average normalized birth rates increased (**C**) and, concomitant with this, average individual energy decreased (**D**).

We next analyzed how higher levels of predation affected parameters related to population density. In the model, density is defined as the current population size divided by the population size modifier, N, and affects food availability and predation probability. Under classical conditions, increased extrinsic mortality reduced population size (Fig. 5A), enlarged the total pool of energy that could be foraged (Fig. 5B), increased the birth rate (Fig. 5C), and decreased the average energy reserves of mature individuals (Fig. 5D). Furthermore, we show that the per-individual probability of being predated on average increased with increasing predation modifier (Fig. S5A). Together these findings suggest that, although extra energy is made available to survivors of higher predation, the average individual has less energy and energy is predominantly used for reproduction, leading to faster population turnover.

**Figure 6:**
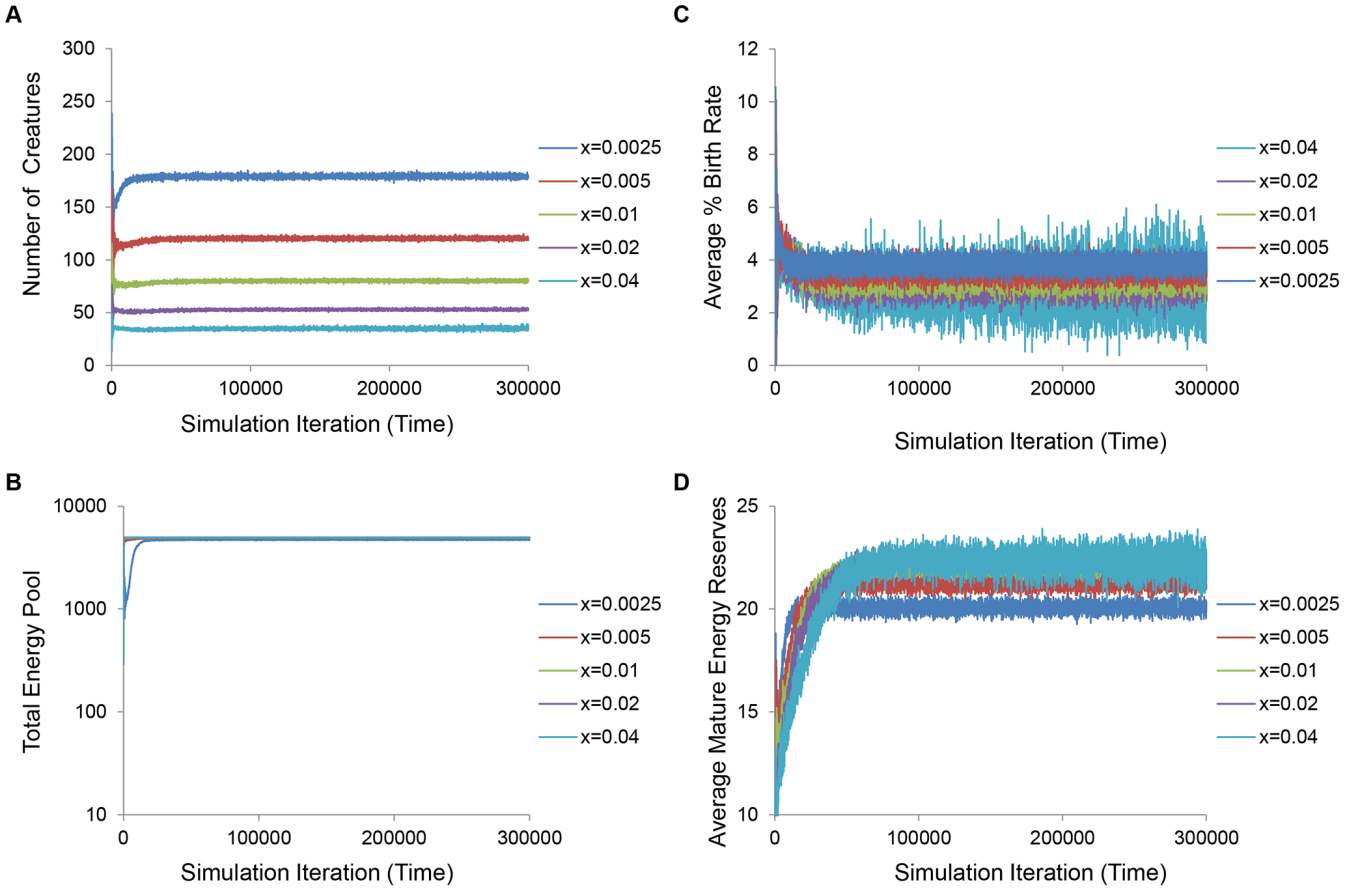
Higher predation impacts parameters related to density dependence under non-classical conditions. Akin to classical conditions, higher predation under non-classical conditions resulted in smaller population sizes (**A**) and a large shared energy pool (**B**). Unlike classical conditions, however, average normalized birth rates decreased (**C**) and the average mature individual energy was increased (**D**).

Under non-classical conditions where individuals on average evolve longer lifespans in response to increased extrinsic mortality, population size also decreased dramatically in response to greater extrinsic mortality (Fig. 6A) and the shared energy pool was never depleted (Fig. 6B). The birth rate decreased (Fig. 6C), and interestingly, the average energy of mature individuals increased with higher predation (Fig. 6D). Thus, under non-classical conditions, survivors of higher predation populations devoted less energy into reproduction and enjoyed increased energy levels, despite the aforementioned extra investments in somatic maintenance. In addition, the evolution of energy investment away from maturation actually resulted in a decreased average probability of predation with increasing predation modifier (Fig. S5B). These are the key differences between the classical and non-classical outcomes, as survivors of conditions leading to a classical decrease in maintenance had less energy under higher levels of predation and were on average more likely to die from predation during each simulation iteration.

## Discussion

It is becoming increasingly evident that classical predictions of aging cannot always explain trends observed in natural populations, with individuals from some natural populations exhibiting longer lifespans in response to increased extrinsic mortality [31]–[34]. Assuming equal susceptibility to extrinsic death factors, and infinite energy, individuals with the highest reproductive capacity (i.e., longest cumulative reproductive lifespan) will outcompete their neighbors and quickly become dominant. However, we know that aging evolved under stochastic energy-limiting conditions with underlying costs associated with faster maturation and somatic repair. Therefore, we decided to start from first principles, developing a relatively simple agent-based energy allocation computational model in which the maturation and intrinsic death times were inherited traits requiring devotion of limited energy resources foraged from a shared energy pool.

Using an unbiased, simulated annealing approach and a stochastic, agent-based model (Fig. 1), we show that increased lifespan can evolve naturally in response to increased extrinsic predation pressure under specific energy-allocation conditions (Fig. 2). We demonstrate that increased extrinsic mortality can have differential effects on evolved lifespan and maturation age (Fig. 3). These changes are accompanied by corresponding reallocations of energy between somatic maintenance and early peak fertility (Fig. 4 and S4). Specifically, our optimization approach revealed that the evolution of decreased average longevity occurs when both food production and mating costs are low (Fig. 2A and 2B). Dissecting the individual energy allocation under these so called “classical” conditions showed that juveniles evolved to invest relatively more energy into maturation compared with somatic maintenance and metabolism (Fig. 4A–C) and mature individuals devoted themselves to reproduction, at the cost of somatic repair (Fig. S4A and S4B). Since reproductive success is tied to the number of energetically costly mating attempts, this would result in earlier and more frequent reproductive success and subsequently a faster population turnover. Although average population size shrinks and the total energy pool for foraging is increased with increasing predation modifier, the average individual has less energy due to the extra investments into reproduction (Fig. 5A–D), and is subject to a higher per-iteration probability of death from predation (Fig. S5A). On the other hand, when optimizing for longevity increases, our simulated annealing approach revealed that longer lifespans naturally evolve when food production is relatively high and when mating incurs a significant energetic investment (Fig. 2C and 2D). Under these so called “non-classical” conditions, energy devoted to longevity is increased solely at the cost of significantly longer maturation times (Fig. 4D–F). Higher levels of predation again decrease population size and increase the total shared amount of resources, but total investments in reproduction evolve to be lower, the average individual has more energy (Fig. 6A–D and Fig. S4C and S4D), and the per-iteration predation probability decreases slightly (Fig. S5B). In other words, since reproduction is prohibitive, individuals require time to amass significant energy reserves before reproducing. To ensure that they do not die from intrinsic causes while they accrue the necessary energy, investment in repair is significantly increased, and investment in fast maturation becomes unnecessary. At the same time, since reproduction becomes less frequent, the overall population remains relatively sparse. This low density actually leads to an overall decrease in per-iteration predation probability, further increasing the benefit of the evolved longer lifespans. Finally, we briefly explore the effect of an increasing fertility pattern by showing that the observed pattern of the evolution of increasing or decreasing longevity holds without the need for reoptimization, when the number of offspring increases with age (Fig. S3).

It was previously shown by Reznick *et al.* that guppies from high-predation localities exhibit delayed senescence as well as enhanced fecundity and swimming performance [23]. Relevant to our model, the authors discuss two theories for how rising predation could select for the evolution of belated senescence. The first is that changes in density regulation may indirectly benefit individuals by creating a surfeit of resources for the survivors. Experimental work has lent credence to this theory, finding that guppies subjected to elevated levels of predation do indeed have, on average, higher per-capita resource availability [28]. This is consistent with our findings, which demonstrate that rising levels of predation decrease population density and increase the total energy pool that can be foraged (Fig. 5 and 6). Moreover, our simulated annealing optimization identified food availability as a critical factor underlying the disparate effects on evolved lifespan, with low and high food turnover being linked to decreased and increased lifespan, respectively (Fig. 2). Survivors from predation enjoyed access to an enlarged energy pool under both increase and classical conditions, however, indicating that resource availability alone is likely not sufficient to drive the evolution of shorter or longer lifespans. Furthermore, a critical difference between classical and non-classical conditions was that the average individual had less energy under classical conditions (Fig. 5D) while the average individual had more energy under non-classical conditions (Fig. 6D). While increased extrinsic mortality ensured that all surviving individuals were always satiated under both types of conditions, a relatively cheap mating cost coupled with the increased investment in earlier maturation actually resulted in lower average energy levels of mature individuals under the classical conditions. On the other hand, non-classical conditions prompted mature individuals to stockpile their energy reserves in “anticipation” of mating.

The second theory discussed is that differences in reproduction may account for how an individual chooses to allocate its energy in response to an extrinsic source of mortality. Most vertebrates, such as mice and humans, exhibit a hump-shaped fecundity curve, peaking sexually shortly after maturation and then suffering a slow, gradual loss in reproductive efficiency [31]. This is not ubiquitous, however, as numerous species have been identified that have anomalous sexual trajectories. The Blanding's turtle, for example, exhibits a low reproductive potential and a reproductive output that increases gradually with age [35]. Naked mole rats suffer no obvious decline in fecundity over time and have atypical, eusocial mating habits centered on a single queen. Violent squabbles between females vying for queen status are not uncommon and can last for weeks. When a new queen takes over, she cannot reproduce until her body undergoes significant changes and, when she does, she will exhibit an exceptionally long gestation period of approximately 70 days [36]–[38]. As such, we would tentatively suggest that “mating costs” or the effort required to mate may be abnormally high in naked mole rats. Interestingly, both the Blanding's turtle and the naked mole-rat are remarkably long-lived for their body sizes and display signs of negligible senescence [30]. Of the three parameters which differed significantly under classical and non-classical conditions, two dealt directly with reproduction – mating energy and energy mating threshold. Mating energy is the energy expended during mating and energy mating threshold is the energy needed to be eligible for mating. Conditions which favored the evolution of shorter lifespans were characterized by cheap mating costs and moderate energy mating thresholds. Comparatively, conditions which favored the evolution of longer lifespans were marked by expensive mating costs and high energy mating thresholds (Fig. 2). Of note, while our model assumes a more typical decline in fecundity with age, the pattern of increased lifespan in response to increased extrinsic mortality under the non-classical conditions also holds if fertility increases with age (Fig. S3). These data demonstrate that, in response to increased predation, the energetic requirements associated with reproduction are a paramount determinant of whether a population will more likely follow the classical or non-classical evolutionary path.

In summary, our agent-based modeling results suggest two evolutionary strategies for dealing with increased age-independent extrinsic mortality: the classical response involving faster maturation and faster aging, and the non-classical response involving delayed maturation and delayed aging. These findings are congruent with and build upon the disposable soma theory, which posits that trade-offs between reproduction and somatic maintenance underlie evolved changes in aging. Furthermore, our results corroborate the wealth of data in opossums [12], Daphnia [13], [14], guppies [23], [28], nematodes [24], flies [15], and other organisms which suggest that extrinsic mortality can drive evolutionary changes in lifespan. The predictions made by our model also have important implications for life-history theory and aging. Increased investment in somatic maintenance is predicted to evolve under increasing extrinsic mortality conditions if mating is considered to be energetically demanding (e.g., long mate search times, maintenance of large territories, or courtship behaviors). Furthermore, the evolved intrinsic death times are expected to be highly variable (Fig. 3F), with significantly longer lifespans expected of a sub-population of individuals (although the shape of the repair cost function may affect the variability). This is likely the case because, as reproduction is relatively costly, significant time would be required to obtain the energy reserves required for mating. Large amounts of resources would help ensure that energy is always available and, despite the high levels of predation, further investments in somatic maintenance would allow these individuals to accumulate the necessary energy before dying of intrinsic causes. At the same time, the reduced population turnover leads to decreased population density and lower predation probabilities (Fig. S5). In other words, animals that live longer can accumulate additional energy reserves and reproduce more than those that do not. Investment in fast maturation therefore becomes expendable and investments in delayed aging take precedence. A similar concept was presented by Shanley and Kirkwood [39]. By using a mathematical life-history model, the authors showed that periods of famine can cause individuals to shift resources away from reproduction and toward somatic maintenance, since juvenile survival was lower when resources were scarce. This allowed individuals to survive famines and, when resources were abundant, avail themselves of the newfound energy and engage in reproduction. The authors theorized that this may be the evolutionary basis of caloric restriction and its lifespan-extending effects [39].

Alternatively, if mating is relatively cheap and if sources of energy are scarce, a better evolutionary strategy is to forgo somatic maintenance in lieu of earlier peak fertility as suggested by classical theories of aging. Since the odds of surviving long enough to exhibit signs of aging are so low, individuals that mature earlier under these classical conditions of high predation will be able to produce more offspring than those that mature at later dates. Since resources are limited and mating is fairly affordable, investments in early fertility, as the mutation accumulation, antagonistic pleiotropy, and disposable soma theories would suggest, take precedence over investments in somatic maintenance. Interestingly, the distribution of intrinsic death times under classical conditions is still expected to be approximately normal or log-normal, with a significant coefficient of variation (Fig. 3E), suggesting that variability in intrinsic death times naturally arises and stabilizes in populations. However, this variability is still relatively low compared to the variability in death ages that evolved under non-classical conditions (Fig. 3F). Particularly supportive of the disposable soma theory, both our classical and non-classical conditions reveal a trade-off between somatic maintenance and reproduction. Our model also shows, however, that the paradigmatic prediction regarding increased extrinsic mortality is incomplete, as additional factors can dictate the evolutionary response to higher rates of externally-induced death.

In addition, our results suggest that increased extrinsic mortality lead to a higher probability of population extinction in a non-linear fashion under non-classical conditions (Fig. S2B). However, under classical conditions, high predation (x = 0.04) lead to stable simulations in over 99% of the 400 independent simulations. While an in-depth analysis of population stability in the context of the evolution of aging is outside the scope of this work, it is possible that the classical response to extrinsic mortality pressures results in a more stable long-term solution, compared to the “slow-and-steady” non-classical response, which works by investing in the individual. This may be especially true during periods of highly fluctuating environmental conditions (as reviewed in [40]). Similarly, we notice that the force of selection declines as the population intrinsic death and maturation times approach their respective equilibrium population distributions (Fig. 3 E,F). This can be readily seen by the rapid decrease in the rate of change of population average statistics with simulation iteration (i.e., mean T_die_ and T_mat_ in Fig. 3 A–D). This rate differs between the classical and non-classical conditions (equilibration by ∼50,000 iteration in the classical cases as seen in Fig. 3A,C, and longer for the non-classical case as seen in Fig. 3B,D), highlighting the increased “evolvability” of population under classical conditions [41]. Fluctuations about the equilibrium point are also noticeably higher in the non-classical case. This can be explained in part by the shape of the T_die_ population distributions, which are narrowly distributed about a mean value for the classical case (Fig. 3E), but highly skewed in the non-classical case at higher values of the predation modifier (Fig. 3F). This implies that in the non-classical case, individuals in the model are subject to varying selective forces, which may allow for higher population robustness in the face of very rapid changes, and further stresses heterogeneous modeling approaches.

While our modeling approach includes parameters for maturation, mating, metabolism, and maintenance and is able to capture population heterogeneity and stochastic effects, many parameters still remain to be explored in future studies. For example, seasonal fluctuations in shared energy generation may further affect the evolution of non-classical responses to increased extrinsic mortality. Also, for simplicity we assumed that extrinsic mortality is not age-dependent, although certain age groups may be more susceptible to predation than others [25], [27], [42]. Likewise, individuals in a population may not be equally susceptible to an extrinsic mortality source. Pathogens, for example, are less of a mortality hazard for individuals with strong immune systems than those with weaker immune systems. Although some individuals may be more capable of dodging predators than others, predation is likely to affect individuals more haphazardly than pathogens [43]. Moreover, in an antagonistic pleiotropy model developed by Williams and Day, the authors demonstrate that selection against physiological deterioration in response to interactive mortality sources can vary with age as well as an individual's internal condition [27]. Relevant to this, heat stress in nematodes engenders the evolution of longer lifespans [24]. Heat stress likely selects for individuals that have abnormally high thermotolerance and this stress resistance may be coupled to other factors that promote longevity. These factors, in addition to the factors we incorporate in our model, could introduce another level of complexity and detail to future analyses.

Ideally, our theoretical findings would be validated experimentally be empiricists. For example, key predictions of our model are that resource availability and reproductive costs largely determine how aging evolves in response to increased predation. Our model optimization predicts that longer lifespans will evolve in the presence of relatively high resource availability and when mating is relatively costly. In contrast, we find that shorter lifespans evolve when food is relatively scarce and reproduction is relatively cheap. Longer lifespans are associated with later maturation ages and, conversely, shorter lifespans are associated with earlier maturation ages. Numerous studies could be designed to test these predictions. For example, we predict that populations growing under food-limiting conditions are more likely to evolve shorter intrinsic lifespans in response to increased random mortality, if the cost of reproduction were relatively cheap. On the other hand, we would predict that a population of individuals that must invest a significant proportion of their energy into acquiring mates (e.g., development of sexually attractive characteristics, courtship, or mate search) to evolve longer lifespans if food is plentiful and the environment is relatively stable. While controlling food availability is relatively straight forward, various strategies could be employed to adjust the costs associated with mating. Mutations known to enhance or impede reproduction could be introduced or, alternatively, populations could be grown in such a way that the probability of male and female interaction is greatly reduced and/or requires higher energetic costs (e.g., by increasing the mate search time). Organisms with different mating efficiencies could also be collated in response to increased extrinsic mortality.

Aging is a multifarious process marked by epigenetic alterations, telomere shortening, genomic instability, exhaustion of stem cells, defective proteostasis, and other complex and interacting factors. It is also believed to underlie a plethora of age-related ailments such as cancer, Alzheimer's disease, and diabetes [44]–[47]. The evolutionary theories of aging have guided aging research for decades and shape how we view senescence as well as the feasibility of therapeutic interventions for age-related damage [48]. Further efforts at demystifying the evolutionary basis of this phenomenon are therefore critical to truly understanding its underlying mechanisms as well as for developing preventative and rejuvenative treatments for its associated ailments.

## Supporting Information

Figure S1
**Graphical depictions of various repair cost functions over time.** The shape of the repair function was allowed to change during the simulated annealing optimization. The repair function determines the per-iteration energy cost for maintaining a specific 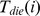. Per-iteration repair energy costs are shown for “low” and “high” sigmoidal, asymptotic, and linear cost repair functions as detailed in eq. (8).(TIF)

Figure S2
**Different values of predation modifier more commonly caused population extinction then others.** The probability of survival, as a function of time, is shown for differently predated populations under classical (**A**) and non-classical (**B**) conditions (n = 400). Under classical conditions, a value of 0.02 for predation modifier, 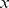, was most correlated with extinction events (**A**). Under non-classical conditions, a predation modifier of 0.04, followed by 0.02, was most commonly associated with population extinction.(TIF)

Figure S3 **Classical and non-classical effects were qualitatively unchanged when fertility increased with age.** To test if an increasing fertility with age could lead to the evolution of increased lifespans, simulations were carried out where individual probability of successful mating was age-independent, but the number of offspring increased linearly with age post-maturation. Average (n = 400) final population average T_mat_ and T_die_ values are shown assuming classical (**A,B**), and non-classical (**C,D**) conditions.
(TIF)

Figure S4
**Changes in evolved lifespan and maturation age are accompanied by corresponding shifts in post-juvenile (mature) energetic investments.** Under classical (**A and B**) and non-classical conditions (**C and D**), the amount of energy devoted to somatic maintenance and reproduction by mature individuals is shown. Under classical conditions, rising levels of predation caused individuals to invest less in somatic maintenance (**A**) and more into reproduction (**B**). Under non-classical conditions, larger values of predation modifier, 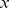, caused individuals to devote more energy toward somatic maintenance (**C**). Investments in reproduction decreased with increasing values of predation modifier, 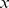 (**D**).(TIF)

Figure S5 **Per-iteration predation rates evolved under classical and non-classical conditions.** Populations subjected to increased extrinsic mortality evolved increasing (**A**) or decreasing (**B**) per-iteration probability of death by predation under classical or non-classical conditions, respectively. Graphs show the per-iteration probability of predation as a function of predation modifier, x, and is normalized for population density.
(TIF)

## References

[1] Kirkwood TB (2002) Evolution of ageing. Mechanisms of ageing and development 123: 737–745. 11869731 10.1016/s0047-6374(01)00419-5

[2] Kirkwood TB, Melov S (2011) On the programmed/non-programmed nature of ageing within the life history. Current biology : CB 21: R701–707. 21959160 10.1016/j.cub.2011.07.020

[3] Ljubuncic P, Reznick AZ (2009) The evolutionary theories of aging revisited–a mini-review. Gerontology 55: 205–216. 19202326 10.1159/000200772

[4] Medawar PB (1952) An unsolved problem of biology. London,: Published for the college by H. K. Lewis. 24 p. p.

[5] Austad SN (1997) Comparative aging and life histories in mammals. Experimental gerontology 32: 23–38. 9088899 10.1016/s0531-5565(96)00059-9

[6] Buffenstein R (2008) Negligible senescence in the longest living rodent, the naked mole-rat: insights from a successfully aging species. Journal of comparative physiology B, Biochemical, systemic, and environmental physiology 178: 439–445. 10.1007/s00360-007-0237-5 18180931

[7] Finch CE, Austad SN (2011) Blind cave salamanders age very slowly: a new member of Methuselah's Bestiary. BioEssays : news and reviews in molecular, cellular and developmental biology 33: 27–29. 10.1002/bies.201000111 21031431

[8] Nussey DH, Froy H, Lemaitre JF, Gaillard JM, Austad SN (2013) Senescence in natural populations of animals: widespread evidence and its implications for bio-gerontology. Ageing research reviews 12: 214–225. 22884974 10.1016/j.arr.2012.07.004 PMC4246505

[9] Williams GC (1957) Pleiotropy, natural selection, and the evolution of senescence. Evolution 11: 398–411.

[10] Kirkwood TB (1977) Evolution of ageing. Nature 270: 301–304. 593350 10.1038/270301a0

[11] Kirkwood TB, Holliday R (1979) The evolution of ageing and longevity. Proceedings of the Royal Society of London Series B, Containing papers of a Biological character Royal Society 205: 531–546. 10.1098/rspb.1979.0083 42059

[12] Austad SN (1993) Retarded Senescence in an Insular Population of Virginia Opossums (Didelphis-Virginiana). Journal of Zoology 229: 695–708.

[13] Dudycha JL, Tessier AJ (1999) Natural genetic variation of life span, reproduction, and juvenile growth in Daphnia. Evolution 53: 1744–1756. 28565448 10.1111/j.1558-5646.1999.tb04559.x

[14] Dudycha JL, Hassel C (2013) Aging in sexual and obligately asexual clones of from temporary ponds. Journal of plankton research 35: 253–259. 23467752 10.1093/plankt/fbt008 PMC3589896

[15] Stearns SC, Ackermann M, Doebeli M, Kaiser M (2000) Experimental evolution of aging, growth, and reproduction in fruitflies. Proceedings of the National Academy of Sciences of the United States of America 97: 3309–3313. 10716732 10.1073/pnas.060289597 PMC16235

[16] Robert KA, Bronikowski AM (2010) Evolution of senescence in nature: physiological evolution in populations of garter snake with divergent life histories. The American naturalist 175: 147–159. 10.1086/649595 20050804

[17] Tozzini ET, Dorn A, Ng'oma E, Polacik M, Blazek R, et al (2013) Parallel evolution of senescence in annual fishes in response to extrinsic mortality. BMC evolutionary biology 13: 77. 23551990 10.1186/1471-2148-13-77 PMC3623659

[18] Austad SN (2010) Methusaleh's Zoo: how nature provides us with clues for extending human health span. Journal of comparative pathology 142 Suppl 1: S10–21. 19962715 10.1016/j.jcpa.2009.10.024 PMC3535457

[19] Shattuck MR, Williams SA (2010) Arboreality has allowed for the evolution of increased longevity in mammals. Proceedings of the National Academy of Sciences of the United States of America 107: 4635–4639. 20176952 10.1073/pnas.0911439107 PMC2842055

[20] Drenos F, Kirkwood TB (2005) Modelling the disposable soma theory of ageing. Mechanisms of ageing and development 126: 99–103. 15610767 10.1016/j.mad.2004.09.026

[21] Cichon M (1997) Evolution of longevity through optimal resource allocation. Proceedings of the Royal Society B-Biological Sciences 264: 1383–1388.

[22] Kramer BH, Schaible R (2013) Life span evolution in eusocial workers–a theoretical approach to understanding the effects of extrinsic mortality in a hierarchical system. PloS one 8: e61813. 23596527 10.1371/journal.pone.0061813 PMC3626611

[23] Reznick DN, Bryant MJ, Roff D, Ghalambor CK, Ghalambor DE (2004) Effect of extrinsic mortality on the evolution of senescence in guppies. Nature 431: 1095–1099. 15510147 10.1038/nature02936

[24] Chen HY, Maklakov AA (2012) Longer life span evolves under high rates of condition-dependent mortality. Current biology : CB 22: 2140–2143. 23084993 10.1016/j.cub.2012.09.021

[25] Abrams PA (1993) Does Increased Mortality Favor the Evolution of More Rapid Senescence. Evolution 47: 877–887. 28567901 10.1111/j.1558-5646.1993.tb01241.x

[26] Le Cunff Y, Baudisch A, Pakdaman K (2013) How evolving heterogeneity distributions of resource allocation strategies shape mortality patterns. PLoS computational biology 9: e1002825. 23341758 10.1371/journal.pcbi.1002825 PMC3547821

[27] Williams PD, Day T (2003) Antagonistic pleiotropy, mortality source interactions, and the evolutionary theory of senescence. Evolution; international journal of organic evolution 57: 1478–1488. 12940353 10.1111/j.0014-3820.2003.tb00356.x

[28] Bryant MJ, Reznick D (2004) Comparative studies of senescence in natural populations of guppies. The American naturalist 163: 55–68. 10.1086/380650 14767836

[29] Kirkpatrick S, Gelatt CD Jr, Vecchi MP (1983) Optimization by simulated annealing. Science 220: 671–680. 17813860 10.1126/science.220.4598.671

[30] Finch CE (2009) Update on slow aging and negligible senescence–a mini-review. Gerontology 55: 307–313. 19439974 10.1159/000215589

[31] Baudisch A, Vaupel JW (2012) Evolution. Getting to the root of aging. Science 338: 618–619. 23118175 10.1126/science.1226467 PMC3705936

[32] Dowling DK (2012) Aging: evolution of life span revisited. Current biology : CB 22: R947–949. 23174294 10.1016/j.cub.2012.09.029

[33] Mitteldorf J, Pepper JW (2007) How can evolutionary theory accommodate recent empirical results on organismal senescence? Theory in biosciences = Theorie in den Biowissenschaften 126: 3–8. 18087751 10.1007/s12064-007-0001-0

[34] Moorad JA, Promislow DE (2010) Evolution: aging up a tree? Current biology : CB 20: R406–408. 20462482 10.1016/j.cub.2010.03.016

[35] Congdon JD, Nagle RD, Kinney OM, van Loben Sels RC (2001) Hypotheses of aging in a long-lived vertebrate, Blanding's turtle (Emydoidea blandingii). Experimental gerontology 36: 813–827. 11295515 10.1016/s0531-5565(00)00242-4

[36] Clarke FM, Faulkes CG (1997) Dominance and queen succession in captive colonies of the eusocial naked mole-rat, Heterocephalus glaber. Proceedings Biological sciences/The Royal Society 264: 993–1000. 10.1098/rspb.1997.0137 PMC1688532 9263466

[37] Henry EC, Dengler-Crish CM, Catania KC (2007) Growing out of a caste–reproduction and the making of the queen mole-rat. The Journal of experimental biology 210: 261–268. 17210962 10.1242/jeb.02631

[38] Roellig K, Drews B, Goeritz F, Hildebrandt TB (2011) The long gestation of the small naked mole-rat (Heterocephalus glaber Ruppell, 1842) studied with ultrasound biomicroscopy and 3D-ultrasonography. PloS one 6: e17744. 21408185 10.1371/journal.pone.0017744 PMC3049790

[39] Shanley DP, Kirkwood TB (2000) Calorie restriction and aging: a life-history analysis. Evolution; international journal of organic evolution 54: 740–750. 10937249 10.1111/j.0014-3820.2000.tb00076.x

[40] Ovaskainen O, Meerson B (2010) Stochastic models of population extinction. Trends in Ecology & Evolution 25: 643–652. 20810188 10.1016/j.tree.2010.07.009

[41] Goldsmith TC (2008) Aging, evolvability, and the individual benefit requirement; medical implications of aging theory controversies. J Theor Biol 252: 764–768. 18396295 10.1016/j.jtbi.2008.02.035

[42] Caswell H (2007) Extrinsic mortality and the evolution of senescence. Trends in ecology & evolution 22: 173–174. 17287044 10.1016/j.tree.2007.01.006

[43] Bronikowski AM, Promislow DE (2005) Testing evolutionary theories of aging in wild populations. Trends in ecology & evolution 20: 271–273. 16701379 10.1016/j.tree.2005.03.011

[44] de Magalhaes JP (2013) How ageing processes influence cancer. Nature reviews Cancer 13: 357–365. 23612461 10.1038/nrc3497

[45] Johnson AA, Akman K, Calimport SR, Wuttke D, Stolzing A, et al (2012) The role of DNA methylation in aging, rejuvenation, and age-related disease. Rejuvenation research 15: 483–494. 23098078 10.1089/rej.2012.1324 PMC3482848

[46] Lopez-Otin C, Blasco MA, Partridge L, Serrano M, Kroemer G (2013) The hallmarks of aging. Cell 153: 1194–1217. 23746838 10.1016/j.cell.2013.05.039 PMC3836174

[47] Kirkwood TB (2005) Understanding the odd science of aging. Cell 120: 437–447. 15734677 10.1016/j.cell.2005.01.027

[48] Mitteldorf J (2006) How evolutionary thinking affects people's ideas about aging interventions. Rejuvenation research 9: 346–350. 16706667 10.1089/rej.2006.9.346

