# Supplementary figures and images for: Effects of Extrinsic Mortality on the Evolution of Aging: A Stochastic Modeling Approach
Source: PLoS One. 2014 Jan 21;9(1):e86602. doi: 10.1371/journal.pone.0086602 (PMC3897743; doi:10.1371/journal.pone.0086602)

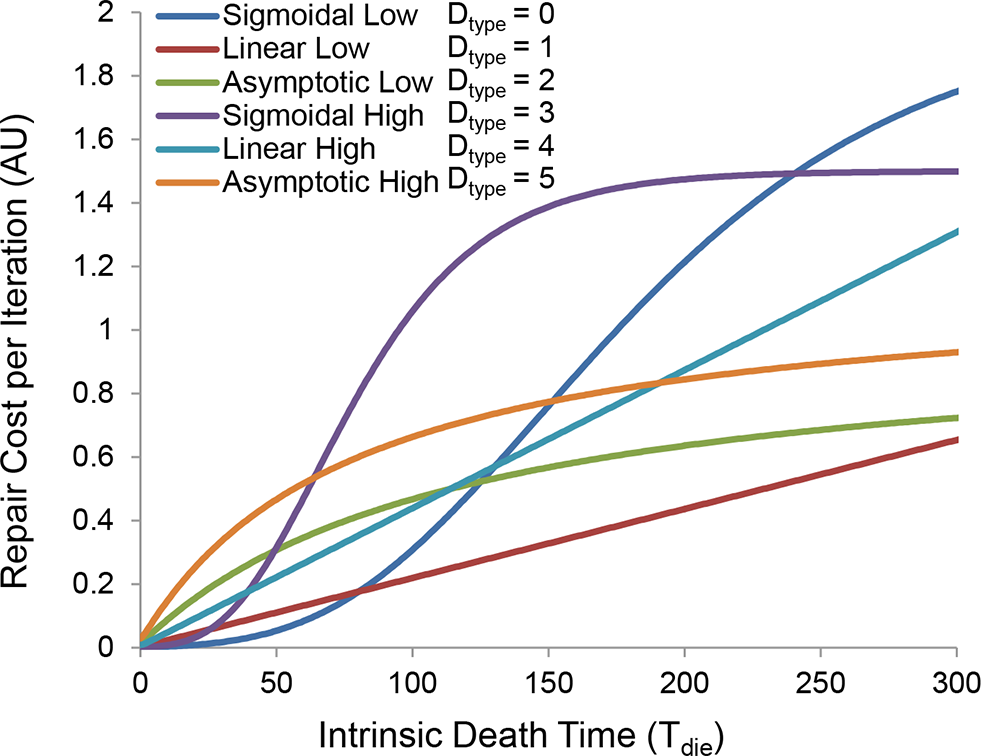

Supplement: Figure S1 — Graphical depictions of various repair cost functions over time. The shape of the repair function was allowed to change during the simulated annealing optimization. The repair function determines the per-iteration energy cost for maintaining a specific . Per-iteration repair energy costs are shown for “low” and “high” sigmoidal, asymptotic, and linear cost repair functions as detailed in eq. (8). (TIF) [file pone.0086602.s001.tif]

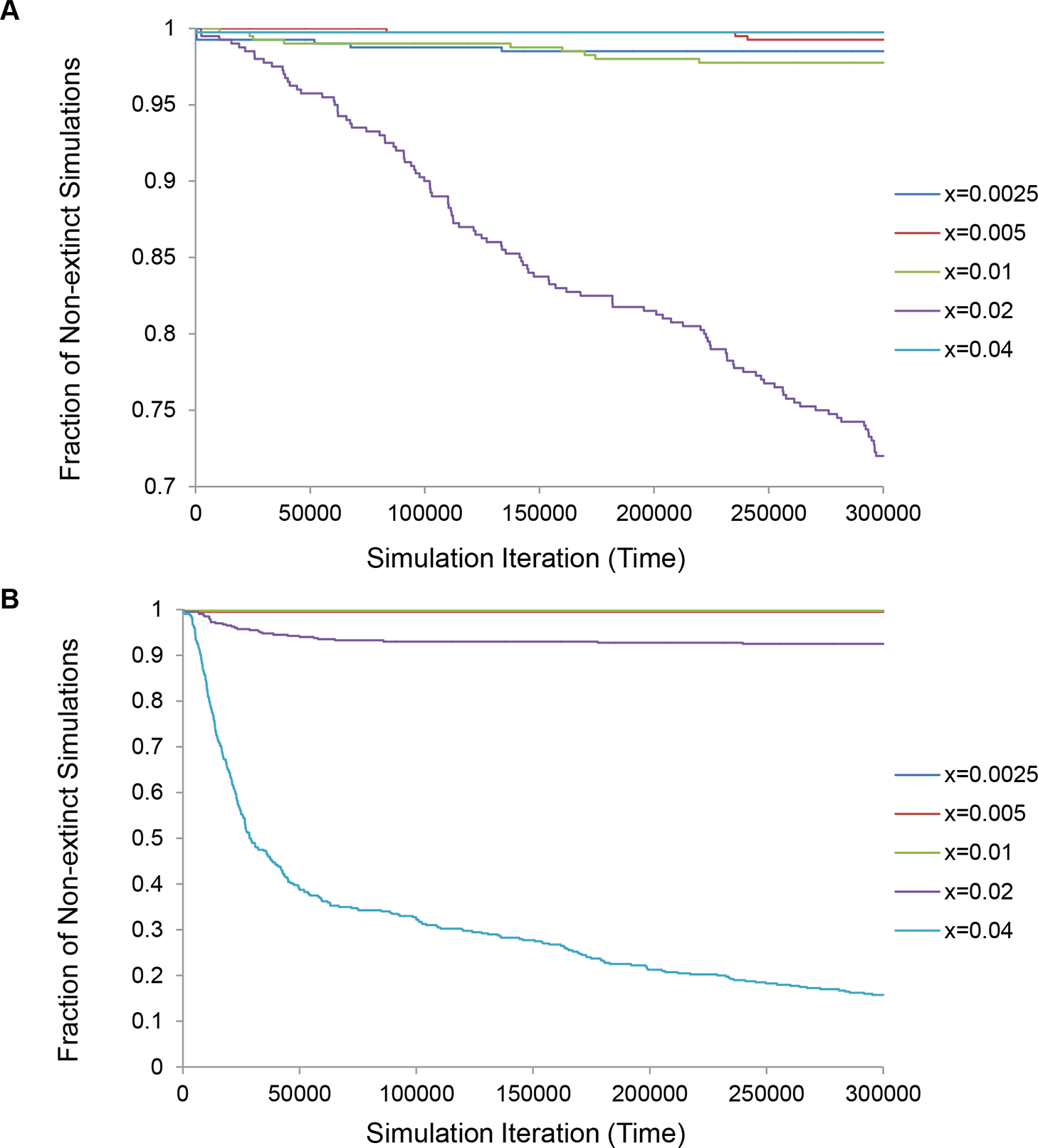

Supplement: Figure S2 — Different values of predation modifier more commonly caused population extinction then others. The probability of survival, as a function of time, is shown for differently predated populations under classical (A) and non-classical (B) conditions (n = 400). Under classical conditions, a value of 0.02 for predation modifier, , was most correlated with extinction events (A). Under non-classical conditions, a predation modifier of 0.04, followed by 0.02, was most commonly associated with population extinction. (TIF) [file pone.0086602.s002.tif]

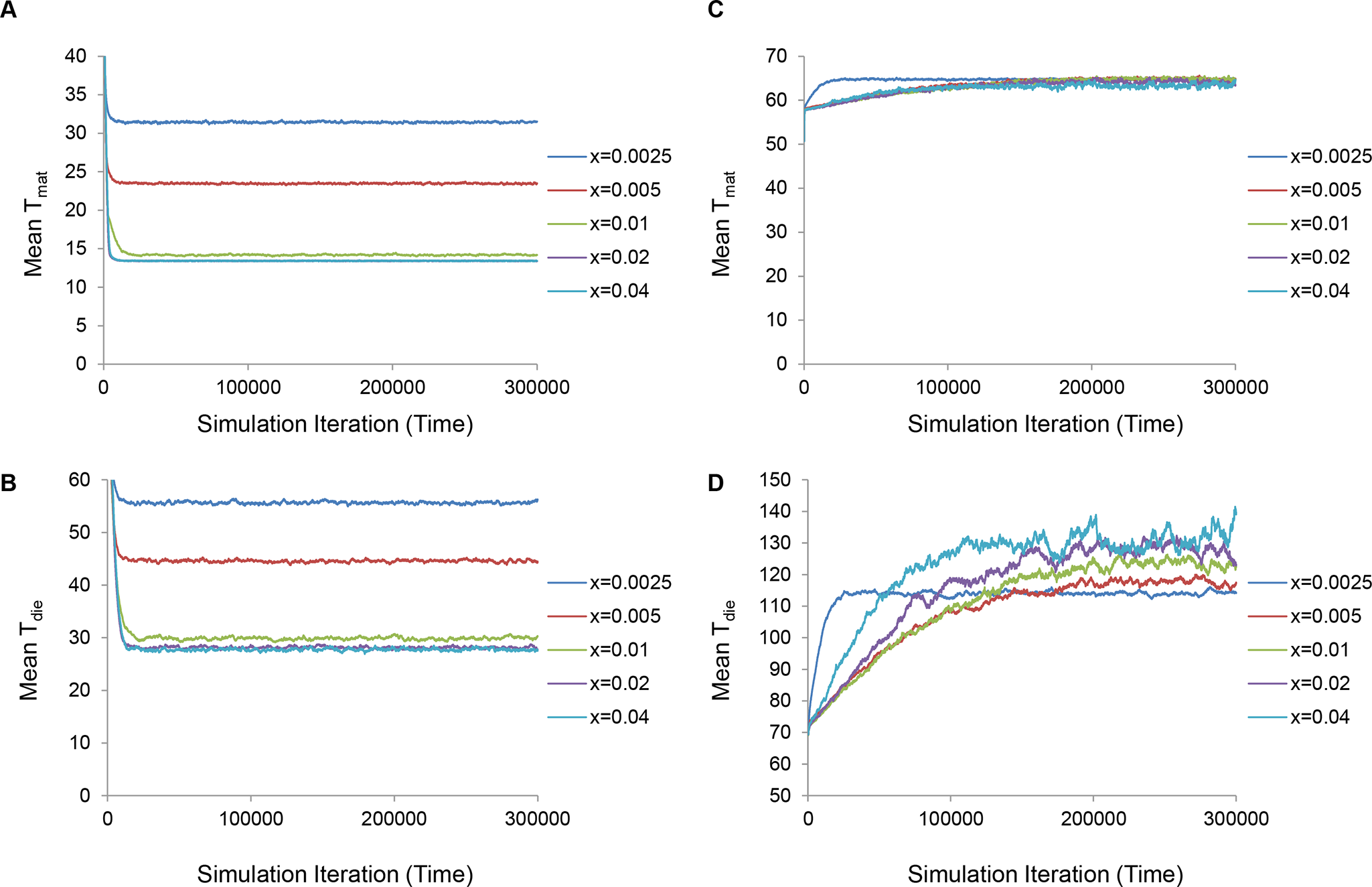

Supplement: Figure S3 — Classical and non-classical effects were qualitatively unchanged when fertility increased with age. To test if an increasing fertility with age could lead to the evolution of increased lifespans, simulations were carried out where individual probability of successful mating was age-independent, but the number of offspring increased linearly with age post-maturation. Average (n = 400) final population average Tmat and Tdie values are shown assuming classical (A,B), and non-classical (C,D) conditions. (TIF) [file pone.0086602.s003.tif]

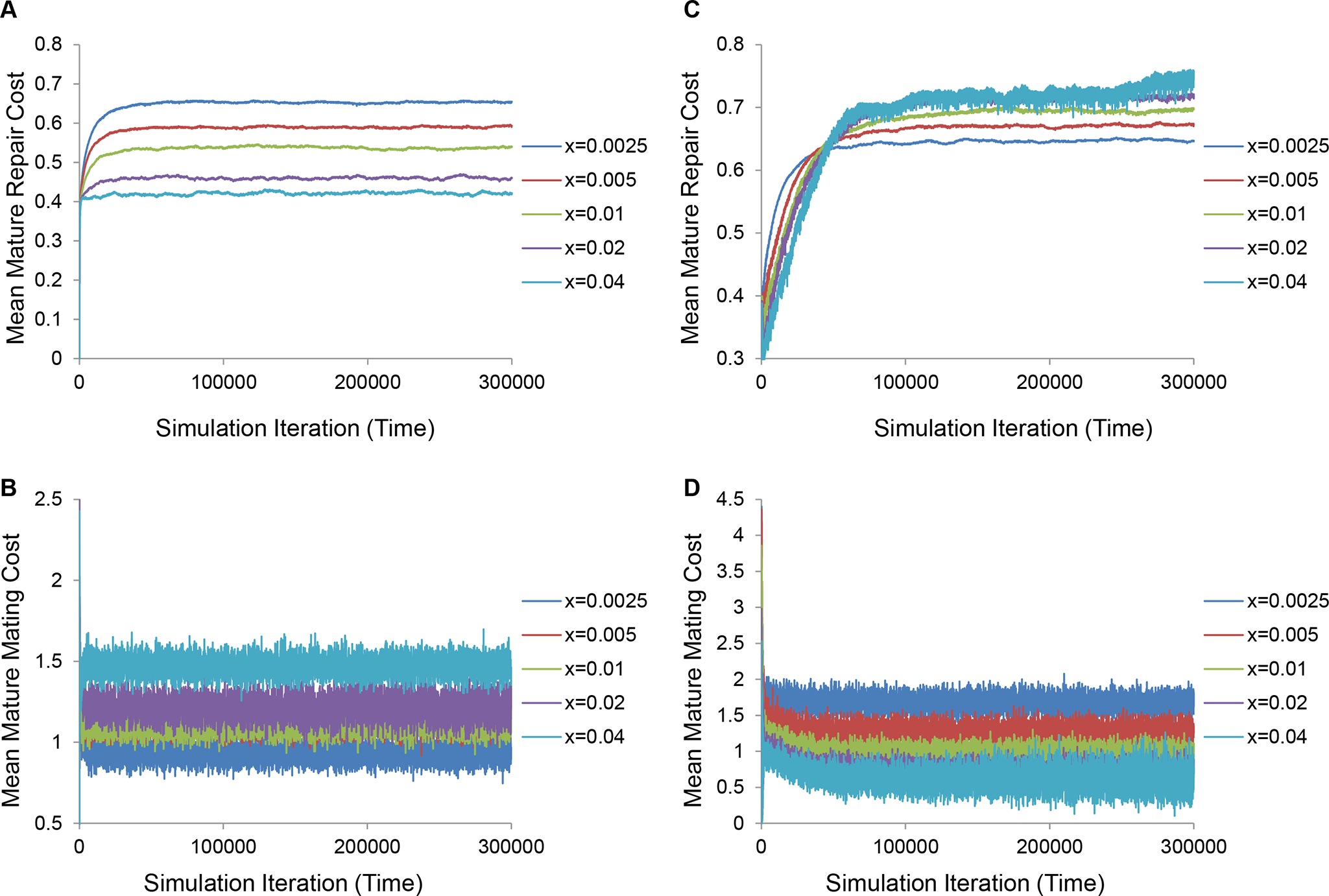

Supplement: Figure S4 — Changes in evolved lifespan and maturation age are accompanied by corresponding shifts in post-juvenile (mature) energetic investments. Under classical (A and B) and non-classical conditions (C and D), the amount of energy devoted to somatic maintenance and reproduction by mature individuals is shown. Under classical conditions, rising levels of predation caused individuals to invest less in somatic maintenance (A) and more into reproduction (B). Under non-classical conditions, larger values of predation modifier, , caused individuals to devote more energy toward somatic maintenance (C). Investments in reproduction decreased with increasing values of predation modifier, (D). (TIF) [file pone.0086602.s004.tif]

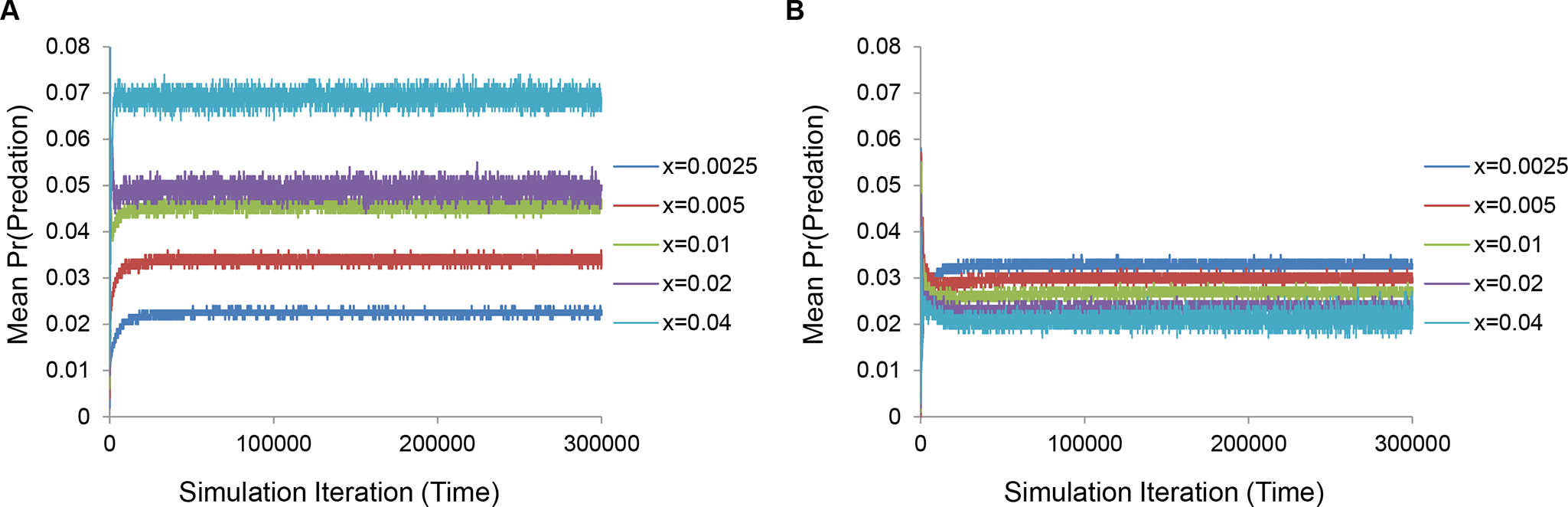

Supplement: Figure S5 — Per-iteration predation rates evolved under classical and non-classical conditions. Populations subjected to increased extrinsic mortality evolved increasing (A) or decreasing (B) per-iteration probability of death by predation under classical or non-classical conditions, respectively. Graphs show the per-iteration probability of predation as a function of predation modifier, x, and is normalized for population density. (TIF) [file pone.0086602.s005.tif]
